# The effects of enteric-coated **s**odium bicarbonate supplementation on 2 km rowing performance in female CrossFit® athletes

**DOI:** 10.1007/s00421-023-05140-4

**Published:** 2023-01-27

**Authors:** Robyn A. X. J. Martin, Nathan P. Hilton, S. Andy Sparks, Bryan Saunders, Lars R. McNaughton

**Affiliations:** 1 Sport Performance, Exercise and Nutrition Research Group, Department of Sport and Physical Activity, Edge Hill University, Ormskirk, L39 4QP Lancashire UK; 2 Applied Physiology & Nutrition Research Group, University of São Paulo, São Paulo, Brazil

**Keywords:** Extracellular Buffer, Acid–base balance, Time trial, Gastrointestinal symptoms

## Abstract

**Purpose:**

Sodium bicarbonate (SB) supplementation can improve exercise performance, but few studies consider how effective it is in female athletes. The aim of the study was to establish the effect of individually timed pre-exercise SB ingestion on 2 km rowing time trial (TT) performance in female athletes.

**Methods:**

Eleven female CrossFit® athletes (mean ± SD age, 29 y ± 4 y, body mass, 64.5 kg ± 7.1 kg, height, 1.7 m ± 0.09 m, peak oxygen uptake [VO_2peak_], 53.8 ± 5.7 mL·kg^−1∙^min^−1^). An initial trial identified individual time-to-peak [HCO_3_^−^] following enteric-coated 0.3 g·kg^−1^ BM SB ingestion_._ Participants then completed a 2 km TT familiarisation followed by a placebo (PLA) or SB trial, using a randomised cross-over design.

**Results:**

The ingestion of SB improved rowing performance (514.3 ± 44.6 s) compared to the PLA (529.9 ± 45.4 s) and FAM trials (522.2 ± 43.1 s) (*p* = 0.001, *p*η^2^ = 0.53) which represents a 2.24% improvement compared to the PLA. Individual time-to-peak alkalosis occurred 102.3 ± 22.1 min after ingestion (range 75–150 min) and resulted in increased blood [HCO_3-_] of 5.5 ± 1.5 mmol⋅L^−1^ (range = 3.8–7.9 mmol⋅L^−1^). The change in blood [HCO_3-_] was significantly correlated with the performance improvement between PLA and SB trials (*r* = 0.68, *p* = 0.020).

**Conclusions:**

Ingesting a 0.3 g·kg^−1^ BM dose of enteric-coated SB improves 2 km rowing performance in female athletes. The improvement is directly related to the extracellular buffering capacity even when blood [HCO_3-_] does not change ≥ 5.0 mmol⋅L^−1^.

## Introduction

During high-intensity exercise, fatigue refers to the abrupt deterioration of exercise performance, a gradual increase in perceived exertion which inevitably leads to an inability to maintain the intended magnitude of intensity (Ament and Verkerke et al. 2009). Performance of high-intensity exercise, which demands a large contribution from anaerobic metabolic pathways is associated with a decrease in muscle and blood pH (Hollidge-Hovart et al. 1999). Peripheral fatigue during such high-intensity exercise bouts has been attributed to perturbations in intramuscular homeostasis due to an accumulation of H^+^. Although the mechanisms through which the loss of muscular force across the neuromuscular junction operates are ambiguous (Westerblad 2016), metabolic acidosis is correlated with diminished muscle contractility (Matson and Tran 1993), excitability (Sostaric et al. 2006), as well as the allosteric inhibition of glycolytic enzymes, reducing the rate at which ATP is synthesised (Messonnier et al. 2007). As a result, preventative methods, such as the administration of exogenous buffers that enhance the blood bicarbonate buffering system may be useful in reducing local acid–base disturbances in the exercising muscle.

In an attempt to delay the deleterious effects of fatigue on sport and exercise performance, nutritional strategies have previously been utilised. During single bouts of high-intensity exercise of ~ 1 to 10 min in duration (Gough et al. 2017a, 2017b; McNaughton 1992a) or repeated bouts of short-duration (4–90 s) exercise (Miller et al. 2016), inducing metabolic alkalosis by means of sodium bicarbonate (SB) ingestion, has been found to optimise numerous performance criteria. The use of SB supplementation can augment buffering capacity and robust ion handling by increasing extracellular bicarbonate ion concentration (HCO_3_^−^) (Carr et al. 2011a, b), which are conducive to improved exercise performance. McNaughton (1992a) was one of the first to demonstrate that administration of a 0.3 g·kg^−1^ body mass (BM) dose of SB was effective whilst limiting some of the possible side effects compared to a 0.4 g·kg^−1^ BM dose. Since then, 0.2–0.4 g·kg^−1^ BM has been widely employed in SB ingestion studies for high-intensity exercise performance (McNaughton et al. 2016). Though gastrointestinal (GI) complaints have proven to be ergolytic (Deb et al. 2018), related studies have been successful in eliciting an improvement in exercise performance despite GI distress (Price and Simons 2010). Nevertheless, to ameliorate GI symptoms, novel ingestion strategies have been evaluated, such as the delivery of SB in enteric-coated capsules (Hilton et al. 2020); such ingestion strategies are able to alleviate GI symptoms without hindering the magnitude of induced alkalosis (Hilton et al. 2020).

Although the extant research highlights the efficacy of SB as an effective buffering agent, a recent umbrella review of meta-analyses (Grgic et al. 2017) determined that > 77% of participants in the existing research literature were male. It is, therefore, reasonable to suggest that the generalizability of such literature is skewed, with one meta-analyses declaring it impossible to determine a precise estimate of the pooled effect size for performance (Saunders et al. 2021); only 20% of studies used female participants, with only 7.4% providing group analyses exclusively on women. In general, women’s exercise physiology nutritional supplementation research is inadequate (Burke 2017). Although female athletes possess physiological factors that are likely to diminish high-intensity exercise performance, such as reduced glycolytic enzyme activity (Green et al. 1984), less overall muscle mass (Janssen et al. 2000) and type II muscle fibre distribution (Simoneau and Bouchard 1989), there is unanimity among the data sets, whilst modest, that SB supplementation has a positive ergogenic effect on exercise performance and significant perturbations to blood [HCO_3_^−^] (Saunders et al. 2021). Despite this, there is plausible disparity in the abundance of literature available using male and female athletes/participants making recommendations “research informed”, rather than “research evidenced” for the use of this ergogenic aid.

This study, therefore, investigated the effect of SB ingestion on female performance in CrossFit® athletes using a time-to-peak ingestion protocol. It was hypothesised that an acute loading ingestion protocol of SB would induce metabolic alkalosis and improve 2 km time trial (TT) performance when performed at individual time-to-peak alkalosis.

## Methods

Eleven female CrossFit athletes took part in this study (mean ± SD age, 29 y ± 4 y, body mass, 64.5 kg ± 7.1 kg, height, 1.7 m ± 0.09 m, peak oxygen uptake [VO_2peak_], 53.8 ± 5.7 mL·kg^−1^·min^−1^). All participants regularly participated in CrossFit® for 2.5 ± 0.8 y and completed 3–5 sessions·wk^−1^ for at least 2 years. Participants were free of GI-related disorders, and those with hypertension, renal impairment, or following a salt-restricted diet, were excluded from the study. No participants consumed nutritional supplements or medication at the time of the study (Hilton et al. 2020). The study was approved by the Departmental Ethics Committee (SPA-REC-2021-118.), and participants provided written and verbal consent before taking part in the study. In a double-blind, placebo-controlled, randomised crossover design, after baseline testing and familiarisation, each participant completed a total of three exercise trials, separated by a minimum of 3 days, there were an initial familiarisation (FAM) trial followed by two experimental trials performed in a randomised order. Each trial consisted of performing a 2 km rowing TT after consuming either, no supplement (FAM), a capsule number and taste matched placebo of 0.07 g^.^kg^−1^ BM sodium chloride (PLA), or 0.3 g^.^kg^−1^ BM enteric-coated SB.

During visit one, participants completed a maximal cardiopulmonary exercise test to determine VO_2_peak. Individual responses to SB ingestion were established throughout the second visit to dictate the subsequent pre-exercise ingestion times (TTP) for the rowing time trials. To minimise the confounding effects of nutritional intake on exercise performance, participants were required to abstain from alcohol consumption and vigorous exercise ≥ 24 h prior to any experimental testing (Hilton et al. 2020), as well as caffeine being prohibited ≥ 12 h before testing. Written nutritional diaries were obtained and replicated in subsequent trials (Gough et al. 2017b). Pre-test nutritional instructions were confirmed verbally with each participant prior to any testing. Experimental trials were also conducted at the same time (Reilly 1990) to account for circadian rhythms, and participants arrived at the laboratory in a 4-h postprandial state.

To determine VO_2peak_, participants undertook an incremental exercise test to volitional exhaustion using an electromagnetically braked cycle ergometer (Lode Sport Excalibur, The Netherlands). Following a 5-min warm-up at 70 W and at a self-selected cadence of 70–120 r⋅min^−1^, the workload was increased by 1 W every 2 s (30 W^.^min^−1^) until volitional exhaustion. A gas analyser (K5, Cosmed, Italy) was used to measure breath-by-breath gases during the test, whilst heart rate and whole-body rates of perceived exertion (RPE) were recorded every 60 s (Borg 1973). A heart rate within 10 b^.^min^−1^ of age-predicted maximum; respiratory exchange ratio (RER) > 1.10 arbitrary units (AU), and RPE > 18 AU were considered confirmation that VO_2_peak had been attained (Midgley et al. 2007).

Given the large inter-individual variability in time-to-peak blood [HCO3^−^] (Sparks et al. 2017), individual responses to ingestion of 0.3 g·kg^−1^ BM of enteric-coated SB capsules (size 00) were determined to allow exercise to be timed with peak [HCO3^−^], which has been shown to be repeatable in males (Gough et al. 2017c). Enteric-coated capsules (VCap®, Lonza, Morristown, NJ, USA) were manually filled with SB (Dr Oetker, Bielfield, Germany) by independent technicians using a manual capsule filling device. Following ingestion, participants remained seated, whilst fingertip capillary blood samples were collected in a 95 μL heparin-coated clinitube every 15 min for 180 min post-ingestion. This sample period was chosen to avoid missing significant absorption characteristics and peak blood [HCO3^−^] (Carr et al. 2011b). Fingertip capillary blood samples were collected using an aseptic technique and analysed immediately (Radiometer, ABL800 basic, Denmark) for blood [HCO_3_^−^] and pH.

On arrival at the laboratory, participants were required to remain seated for 20 min before a pre-ingestion (baseline) capillary blood sample was obtained. Under double-blind, placebo-controlled, randomised crossover conditions, participants were required to ingest either 0.3 g·kg^−1^ BM of SB, or a placebo in enteric-coated capsules; capsule number and taste were matched between the SB and the placebo. After the pre-determined time-to-peak blood [HCO3^−^] has passed, a further blood sample was taken immediately the pre-exercise acid–base balance was assessed with a second blood sample. All blood samples were immediately analysed for [HCO_3_^−^] and pH. The rowing ergometer (Concept 2, Nottingham, UK) drag factor was set to 120 in accordance with a previous 2 km TT and participants completed a 7 min warm-up replicated from a previously published protocol (Carr et al. 2011b). The warmup phase consisted of 4 min at 70% of maximal power output, a subsequent 3 min passive rest period, and 2 × 10 maximal strokes prior to the 2 km TT. Participants were instructed to complete the trial as fast as possible. All visual feedback of distance and performance time were obscured during the TT’s, with verbal notifications of distance covered being provided for each 500 m until 1500 m, thereafter the participants were notified of each 100 m completed. Overall performance time and mean power output were measured as performance criteria. During the TT’s RPE (Borg 1973) was measured every 500 m. Blood parameters were measured prior to ingestion, pre-exercise and immediately after the TT was completed.

### Statistical analysis

Normality testing was completed using a Shapiro–Wilk test on all variables to determine the appropriate statistical test. Performance measures, time to completion, stroke rate and power output and GI symptoms were then subsequently analysed using a one-way analysis of variance (ANOVA) for repeated measures. Blood metabolites ([HCO_3_^−^] and pH), and [La^−^]) were analysed using a two-way (condition x time) ANOVA for repeated measures. Bonferroni-adjusted post-hoc paired comparisons were determined when a significant main effect was reported. Mean RPE and within TT RPE responses were analysed using Friedmans ANOVA. Partial eta squared (pη^2^) was calculated as an effect size for ANOVA and Kendall’s W was used as an effect size for Friedman’s ANOVA. Hedges g was used to determine effect size (ES) and their 95% confidence intervals (CI) for paired comparisons, as it eliminates the positive bias found in Cohens d effect size in sample sizes less than 20 (Lakens et al. 2013). Effect sizes were interpreted as trivial (< 0.20), small (0.20–0.49) moderate (0.50–0.79) and large (≥ 0.80) for pη^2^, and small (0.1–< 0.3), medium (0.3–< 0.5), and large (> 0.5) for Kendall’s W, and as small (0.2), medium (0.5), and large (0.8) for Hedges g. Differences in the GI responses between conditions were analysed using a weighted cases chi squared test (*χ*^2^). Relationships between changes in performance time, change in [HCO_3_^−^] and differences in aggregated GI symptoms were assessed using Spearman’s correlation coefficients. All statistical analysis was undertaken using SPSS statistics editor (v25.0, SPSS Inc, Chicago, USA). Statistical significance was set at *p* ≤ 0.05 with all data reported as mean ± standard deviation.

## Results

**Fig. 1:**
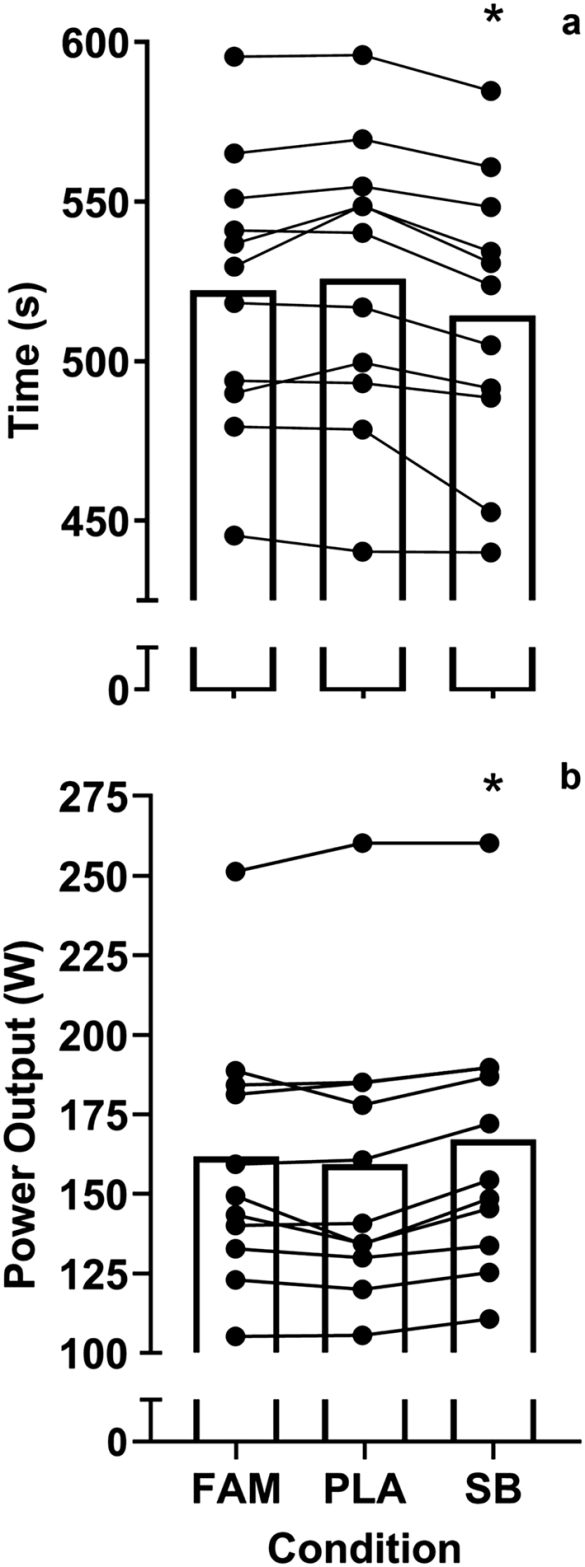
Mean ± SD 2 km TT performance time (**a**) and power output (**b**) during the familiarisation (FAM) and following the ingestion of 0.3 g kg^−1^ BM of sodium bicarbonate (SB) or a placebo (PLA). (*) denotes a significant difference in performance outcome between conditions (*p* < 0.01). Lines represent individual performance responses

The peak [HCO_3-_] was 29.6 ± 1.4 mmol⋅L^−1^ with a range of 28.2–32.7 mmol⋅L^−1^ (Table 1). This represents an absolute change in [HCO_3-_] of 5.5 ± 1.5 mmol⋅L^−1^ (range = 3.8–7.9 mmol⋅L^−1^) and this occurred 102.3 ± 22.1 min after ingestion (range = 75–150 min). In the subsequent exercise performance trials there was a main effect for performance time (*F*_(2,20)_ = 11.40, *p* = 0.001, *p*η^2^ = 0.53), which indicated that ingestion of SB at TTP improved time trial performance compared to the PLA (*p* = 0.001) and FAM trials (*p* = 0.039), representing a moderate effect size (Fig. 1a). This represents a performance improvement of 2.24% when SB was ingested, compared to the PLA. There was no significant difference in performance time between the FAM or the PLA trials (*p* = 0.365) and no trial order effect was observed (*F*_(2,20)_ = 0.26, *p* = 0.769, pη^2 =^ 0.03). Power output (Fig. 1b) was similarly affected by the ingestion of SB (*F*_(2,20)_ = 10.08, *p* = 0.001, *p*η^2^ = 0.50), which allowed participants to produce the highest mean power outputs compared to the PLA (*p* = 0.01) or FAM conditions (*p* = 0.025). Mean power output was not different between the FAM or PLA conditions (*p* = 0.916).

**Table 1:** Individual time to peak and mean (± SD) metabolite responses to 0.3 g·kg^−1^·BM^−1^ sodium bicarbonate ingestion

Blood Metabolites	Participant											Mean ± SD
	1	2	3	4	5	6	7	8	9	10	11	
HCO_3-_ Baseline (mmol⋅L^−1^)	28.7	22.5	24.5	22.2	22.4	24.5	24.6	24.9	24	23.4	23.6	24.1 ± 1.8
HCO_3-_ Peak (mmol⋅L^−1^)	32.7	29.5	28.5	28.2	30.3	28.4	29.7	28.7	30.2	30.8	28.4	29.6 ± 1.4
Absolute Change (mmol⋅L^−1^)	4.0	7.0	4.0	6.0	7.9	3.9	5.1	3.8	6.2	7.40	4.8	5.5 ± 1.5
Time to Peak (min)	75	90	90	90	105	90	90	105	105	150	135	102.3 ± 22.1
Baseline pH	7.38	7.44	7.43	7.37	7.39	7.41	7.43	7.42	7.46	7.44	7.43	7.42 ± 0.03
Peak pH	7.49	7.52	7.46	7.47	7.5	7.46	7.45	7.47	7.48	7.52	7.47	7.48 ± 0.02
Absolute pH Change	0.11	0.08	0.03	0.1	0.11	0.06	0.02	0.06	0.02	0.08	0.04	0.06 ± 0.03
Time to Peak pH (mins)	90	90	45	90	90	175	90	165	105	150	135	111.4 ± 39.6

**Fig. 2:**
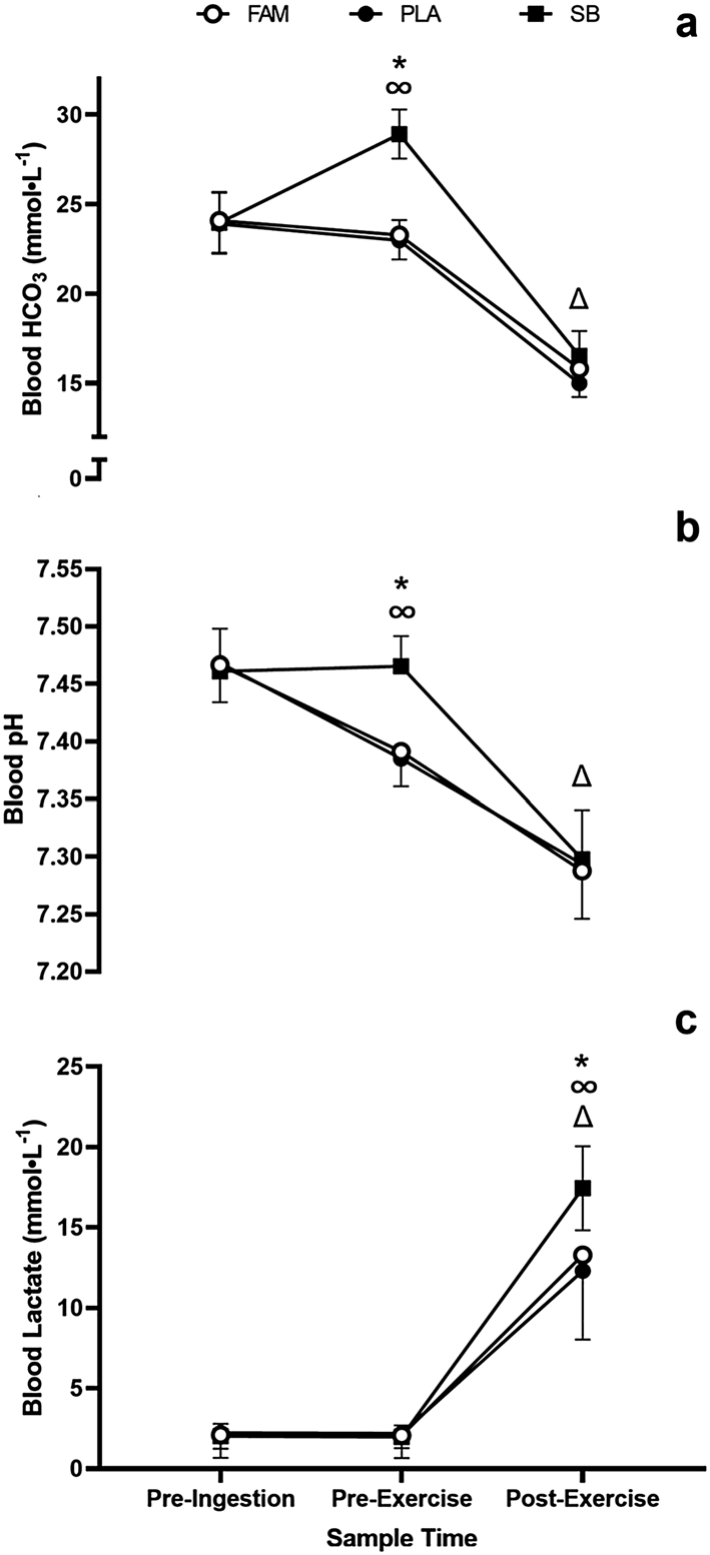
Mean ± SD blood [HCO_3_^−^] (**a**) pH (**b**) and lactate (**c**) responses to the 2 km time trial, during the familiarisation (FAM) and following the ingestion of 0.3 g kg^−1^ BM of sodium bicarbonate (SB) or a placebo. (*) denotes a significant difference between SB and both the FAM and PLA conditions (*p* < 0.01). (Δ) denotes a significant change from the previous sample time (*p* < 0.01). (∞) denotes a condition*time interaction (*p* < 0.001)

During the time trials, the ingestion of SB resulted in significantly altered blood [HCO_3-_] (Fig. 2a) responses (F_(2, 20)_ = 35.27, *p* < 0.0001, *p*η^2^ = 0.78) which represents a large effect. The [HCO_3-_] responses in the FAM and PLA trials were not significantly different (*p* = 0.687) but there was a significant main effect for time (*F*_(2, 20)_ = 198.28, *p* < 0.0001, *p*η^2^ = 0.95) with no change in [HCO_3-_] between pre-ingestion and pre-exercise across all conditions (*p* = 0.205) followed by a significant decrease in [HCO_3-_] on completion of the time trials (*p* < 0.0001). There was, however, a significant Condition*Time interaction effect (*F*_(2, 20)_ = 39.96, *p* = 0.0001, *p*η^2^ = 0.80) caused by the increase in [HCO_3-_] following SB ingestion prior to exercise (MD = 4.96 mmol⋅L^−1^, *t* = 6.87, *p* < 0.0001, *g* = 3.18). The change in performance between PLA and SB was significantly correlated with the change in [HCO_3-_] prior to the start of exercise (*r* = 0.68, *p* = 0.020). Blood pH (Fig. 2b) was significantly decreased following the time trials in all conditions (*F*_(2,20)_ = 96.76, *p* < 0.0001, *p*η^2^ = 0.91) with the same responses occurring in the FAM and PLA trials, but this was not the case in the SB trial (*F*_(2,20)_ = 6.37, *p* = 0.007, *p*η^2^ = 0.39) and this resulted in a significant interaction for condition*time (*F*_(2,20)_ = 8.65, *p* < 0.0001, *p*η^2^ = 0.46) due to the more rapid decrease in pH at the pre-exercise sample time in the FAM and PLA conditions. Blood lactate responses (Fig. 2c) were significantly different as a result of the pre-exercise ingestion strategies (*F*_(2,20)_ = 6.94, *p* = 0.005, *p*η^2^ = 0.41). Over the course of the trials blood lactate was also significantly elevated (*F*_(2,20)_ = 164.5, *p* < 0.0001, *p*η^2^ = 0.94) with different magnitude responses being observed resulting in a main effect for condition*time (*F*_(2,20)_ = 12.32, *p* < 0.0001, *p*η^2^ = 0.55). Following the time trials blood lactate was significantly higher in the SB trial compared to either the FAM (*p* = 0.018) or PLA conditions (*p* = 0.01). The same blood lactate concentrations in the FAM and PLA trials were observed following the time trials (*p* = 0.576). The mean RPE responses (data not presented) were unaffected by the pre-exercise ingestion strategies (χ = 0.00, *p* = 1.00, *w* = 0.00), but there were significant increases in RPE across the duration of the time trials (χ = 31.9, *p* < 0.0001, *w* = 0.97; χ = 30.9, *p* < 0.0001, *w* = 0.94; χ = 29.6, *p* < 0.0001, *w* = 0.90, for FAM, PLA and SB, respectively. The observed post-exercise RPE values of 17.9 ± 1.45, 17.9 ± 2.1 and 18.2 ± 1.1 for FAM, PLA and SB, respectively, were also not affected by the ingestion strategies χ = 0.21, *p* < 0.902, *w* = 0.01.

No gastrointestinal symptoms were observed either prior to ingestion in any of the trials, or at any point throughout the FAM trials. Some GI symptoms were reported in both the PLA and the SB trials, but the frequency of the symptom reports (Table 2) was not significantly different between the PLA and the SB trials (χ^2^ = 0.50, *p* = 0.480). Conversely, the aggregated (symptom severity) total was significantly higher following SB ingestion (χ^2^ = 42.68, *p* < 0.0001). The aggregated GI symptom totals had no influence on the change in performance between PLA and SB (*r* = 0.028, *p* = 0.935), nor was it significantly related to the change in [HCO_3-_] prior to the start of exercise (*r* = 0.221, *p* = 0.513).

**Table 2:** Individual GI symptom scores immediately pre-exercise. Recorded symptoms are displayed in bold, and scores are displayed in parentheses

Participant	Condition		
	FAM	PLA	SB
1	No symptom (0.0)	No symptom (0.0)	**Diarrhoea (7.0), Stomach bloating (8.0)**
2	No symptom (0.0)	No symptom (0.0)	No symptom (0.0)
3	No symptom (0.0)	No symptom (0.0)	No symptom (0.0)
4	No symptom (0.0)	**Nausea (7.0)**	**Bowel Urgency (8.0), Diarrhoea (7.0)**
5	No symptom (0.0)	**Nausea (3.0)**	No symptom (0.0)
6	No symptom (0.0)	No symptom (0.0)	No symptom (0.0)
7	No symptom (0.0)	No symptom (0.0)	**Belching (6.0)**
8	No symptom (0.0)	No symptom (0.0)	No symptom (0.0)
9	No symptom (0.0)	**Bowel Urgency (5.0)**	No symptom (0.0)
10	No symptom (0.0)	No symptom (0.0)	**Bowel Urgency (5.0), Belching (4.0), Diarrhoea (4.0), Stomach bloating (4.0)**
11	No symptom (0.0)	No symptom (0.0)	**Flatulence (2.0), Stomach cramping (4.0), Belching (5.0), Bowel urgency (4.0), Diarrhoea (1.0), Stomach bloating (4.0)**

## Discussion

The present study is the first to investigate the effects of SB ingestion on exercise performance in trained female CrossFit athletes. Furthermore, this study also used the contemporary approach of determining the individualised time to peak alkalosis to optimise the pre-exercise ingestion time whilst also delivering the SB dose using a GI symptom-limiting technique. The key finding was that every participant performed their fastest 2 km rowing TT representing a mean improvement of 2.24% compared to the PLA. The enhanced performance observed following SB ingestion is likely attributable to increases in extracellular buffering capacity, since pre-exercise blood [HCO_3_^−^] and pH were higher following SB ingestion. Enhancing the extracellular buffering capacity in this manner stimulates lactate/H^+^ co-transporters which aid the efflux of H^+^ from the intramuscular space to the extracellular fluid. This enables the enhanced removal of H^+^ and raises the intramuscular pH (Price and Singh 2008). Maximising glycolytic flux is likely to have facilitated the high-intensity exercise during the SB trials, since elevated blood [La-] were observed in such condition post exercise.

Existing literature on the effects of SB on high-intensity exercise protocols have yielded equivocal results with some reporting a positive effect amongst female athletes (Delextrat et al. 2018; McNaughton et al. 1997) and others reporting no effect (Macutkiewicz and Sunderland 2018). These seemingly contradictory findings may in part be explained by the inconsistency of the timing of ingestion in previous studies (Heibel et al. 2018). Optimising the ingestion time has previously been shown to provide a competitive advantage for 2 km rowing TT performance over a generic ingestion time in well-trained athletes (Boegman et al. 2021). In this study (Boegman et al. 2021) the performance times were significantly different between the control (369.0 ± 10.3 s) and the individualised peak (IP) time trial (367.0 ± 10.5 s) [mean difference 1.5 ± 2.4 s (95% CI 0.5 to 2.6 s); *p* = 0.007; *d* = 0.15]. Of the 23 rowers participating, 18 improved their times in the IP trial with 11 participants at or above a 3 s improvement.

Furthermore, using a delivery method such as enteric-coated SB to limit the potential GI symptom severity (Hilton et al. 2020), factors that may be ergolytic following ingestion (Saunders et al. 2014) were likely reduced, providing a more optimal ingestion strategy in the present study. Conversely, more GI symptoms were observed in the placebo and the SB trials in this study compared to previous research using the same ingestion strategy on male cyclists (Hilton et al. 2020). The presence of some GI symptoms in the placebo condition are likely due to the sodium dose which exceeds normal daily intake which may influence osmotic pressure in the intestinal lumen resulting in GI discomfort (Gisolfi et al. 1990). However, the symptoms did not result in worse performance in those most severely affected, nonetheless, the relatively higher ratings in these female athletes warrants further investigation in future work.

In the present study the individual the TTP ranged from 75 to 150 min, which is consistent with enteric-coated SB delivery (Hilton et al. 2020) and the high degree of inter-individual TTP variability, characteristic of [HCO_3_^−^] responses following SB ingestion (Sparks et al. 2017). Whilst there is significant variation between the individual TTP for [HCO_3_^−^] and pH, both variables have been shown to exhibit considerable intra-individual consistency in males (Gough et al. 2017c). The present study used the TTP for [HCO_3_^−^], since this is more repeatable than TTP pH (Gough et al. 2017c), but one clear limitation in the present study is that it is not yet known if the same level of repeatability of the TTP for [HCO_3_^−^] is exhibited in females following the administration of SB. The present study attempted to reduce the time between each trial as much as possible to limit any potential effects of menstrual phase, but the extent to which this may impact the TTP responses is as yet unknown. There was, however, no trial order effect, and every participant performed better in the SB trial compared to the PLA.

Given that the level of alkalosis appears to be dose-dependent, the magnitude of alkalosis, or more specifically, the concentration of [HCO_3_^−^], usually needs to be optimised to elicit maximal ergogenic effects following the ingestion of SB. Previous research suggests, that whilst any increase in [HCO_3_^−^] may strengthen buffering capacity (Jones et al. 2016), a 5 mmol∙L^−1^ increase from baseline is recommended to increase the likelihood of an ergogenic effect (Heibel et al. 2018). Moreover, a rise of 6 mmol·L^−1^ is recommended to identify an absolute ergogenic response (Carr et al. 2011a). Interestingly, in the present study the mean change in [HCO_3_^−^] was 5.5 ± 1.5 mmol·L^−1^, following SB ingestion, with only three of the participants achieving a ≥ 6.0 mmol·L^−1^ response (five participants did not even achieve a ≥ 5.0 mmol·L^−1^ response). There was also a significant correlation between the change in [HCO_3_^−^] and performance improvement between PLA and SB trials, suggesting that the relative change in [HCO_3-_] is related to the ergogenic effect; but that the previously suggested thresholds may not always be required. Indeed, there are likely to be other additional considerations required to determine the success of the SB ingestion on performance than a > 5–6 mmol·L^−1^ change in [HCO_3_^−^], especially when the ingestion strategy is designed to release most of the SB into the gut rather than the stomach. This is in contrast with some of the suggestions by Westerblad (2016), that acidosis has little effect on the causes of skeletal muscle fatigue. Whilst there remain inconsistencies in the findings, (especially due to methodological issues associated with studies using diverse experimental muscle models), nutritional manipulations of extracellular buffering capacity prior to maximal exercise tasks, such that in the present study, provide clear evidence of a fatigue resistance effect. Interestingly, this may not just be peripheral in nature as increased acidosis has recently been shown to have the potential cause central fatigue (Hureau et al. 2022).

Few studies have utilised a female only participant population when investigating SB ingestion and high-intensity exercise performance. This may be due to evidence suggesting that anaerobic exercise performance may be impacted by menstrual cycle phase; with poorer performances being documented typically in the early follicular phase (Materson 1999). The present study did not determine menstrual phase in these athletes, because this represents one of the first steps in determining if there is an ergogenic effect of SB using TTP ingestion and an enteric-coated delivery method. Furthermore, athletes are unlikely to consider menstrual phase as a reason to either not train/compete or take ergogenic aids. However, it is possible that if menstrual phase impacted either absorption, buffering capacity or performance, then the ergogenic effect may be enhanced further if the optimal timing of SB use could be determined in the future, but this remains unclear.

## Conclusion

This is the first study to investigate the effects of enteric-coated SB ingestion on 2 km TT performance in trained female CrossFit® athletes. The main finding is that optimising the timing of the start of exercise to coincide with peak alkalosis, improves TT performance by ~ 2.24% in female athletes. The ingestion of a 0.3 g·kg^−1^ BM dose of SB improved extracellular buffering capacity allowing more intense exercise to be performance. The change in blood [HCO_3_^−^] was related to the performance effect of SB ingestion, but only three athletes’ blood [HCO_3_^−^] increased by ≥ 6.0 mmol·L^−1^ with a further five athletes not reaching a ≥ 5.0 mmol·L^−1^. This suggests that when exercise it timed to coincide with TTP alkalosis using enteric-coated SB delivery methods, the change in blood [HCO_3_^−^] is important, but a threshold of a 5–6 mmol·L^−1^ change is not the only criterion for a likely ergogenic effect.

## Data Availability

The datasets generated during and/or analysed during the current study are available from the corresponding author on reasonable request.

## Abbreviations

ANOVA: Analysis of variance
ATP: Adenosine triphosphate
BM: Body mass
FAM: Familiarisation
GI: Gastrointestinal
HCO_3-_: Bicarbonate anion
La^−^: Lactate anion
P: Placebo
pH: Potential of hydrogen
RER: Respiratory exchange ratio
RPE: Rating of perceived exertion
SB: Sodium Bicarbonate
TT: Time trial
VO_2peak_: Peak oxygen uptake
